# A rigorous theoretical model of fluorescence-based fiber optic sensors: application to D-shaped fibers

**DOI:** 10.1038/s41598-025-33244-8

**Published:** 2025-12-31

**Authors:** Shaghayegh Baghapour, Wen Qi Zhang, Stephen C. Warren-Smith, Sally E. Plush, Shahraam Afshar V.

**Affiliations:** 1 Laser Physics and Photonics Devices Laboratories, School of Physics, Chemistry, and Earth Sciences, Adelaide University, Adelaide, 5095 South Australia Australia; 2 Future Industries Institute, Adelaide University, Adelaide, 5095 South Australia Australia; 3 School of Pharmacy and Biomedical Sciences, Adelaide University, Adelaide, 5000 South Australia Australia

**Keywords:** Optics and photonics, Physics

## Abstract

We develop a comprehensive theoretical model for fluorescence-based fiber optic sensors that accounts for multimodal excitation, incoherent emission from a homogeneously distributed ensemble of individually treated dipole emitters, and multimodal fluorescence capture. Unlike previous models based on bulk excitation and emission, our approach starts from single-dipole physics and extends to a continuous emitter distribution, enabling accurate modeling of spatial interference effects and fluorescence collection. Using this model, a fiber optics sensor, consisting of a D-shaped fiber sandwiched between an input and output fiber, is simulated to investigate the effects of core size, excitation modes, and polishing depth on fluorescence output, thereby identifying configurations that optimize sensor performance. The results indicate that, for fundamental mode excitation, polishing the fiber to approximately halfway through the core diameter enhances the fluorescence output. Additionally, smaller core fibers demonstrate stronger fluorescence output, and excitation with higher-order modes consistently produces greater fluorescence than fundamental mode excitation. This effect is especially pronounced in larger core fibers. These findings suggest that the fluorescence collection limitations in larger core fibers can be mitigated by higher-order mode excitation. Moreover, higher-order mode excitation enables optimal fluorescence output at shallower polishing depths, making it especially advantageous for large-core fibers in practical applications.

## Introduction

Understanding the full optical behavior of fluorescence-based fiber sensors requires a comprehensive model that accurately represents both the excitation and emission processes inside the fiber. In this work, we present a detailed theoretical model that simulates a three-section fiber geometry, comprising excitation, sensing, and collection regions, and uniquely incorporates multimode excitation, emission from an ensemble of individual fluorophores, and multimode fluorescence capture. Our approach treats fluorophores as independent, incoherent dipole sources and analyzes how guided modes interfere to produce spatially varying hotspots in both excitation and capture. These excited fluorophores emit fluorescence omnidirectionally, and the signal is captured across all guided modes in the collection region for delivery to the detector. Unlike previous models that assume bulk excitation and fluorescence emission through the Beer-Lambert law^1–3^, our model is based on a single emitter dipole and then takes the limit of homogeneous coating. This enables its application not only to coating-based sensors, but also to configurations involving isolated or ensemble sources, such as single-photon emitters like nitrogen-vacancy (NV) centers in diamonds^4^.

Fiber optic fluorescence-based sensors show great potential in diverse fields such as medical diagnostics^5^, environmental science^6^, and agricultural monitoring^7^. These sensors are capable of detecting a broad spectrum of chemical ions, including pH^8,9^, hydrogen sulfide^10^, oxygen^11^, biomolecules^12,13^, and gases^14,15^, and physical parameters including temperature^16^. Ongoing research efforts aim to further enhance their performance characteristics, expand their detection capabilities, and explore novel applications in emerging fields such as health monitoring.

Sensing using the distal end of the fiber is found to have limited fluorescence signal collection compared to long length evanescent field sensing, where the entire length of the optical fiber can serve as a sensing element^17^. To improve interaction with the surrounding medium and gain better access to the evanescent field, one common approach is to geometrically modify the fiber^18^. For example, tapering the fiber enhances the evanescent field at the sensing region^14,19^. To date, a variety of tapered optical fiber sensors have been demonstrated to detect biomolecules^19–21^, chemical ions^22^, refractive index^23^, and temperature^24^. Another technique to maximize the evanescent field within the sensing region involves removing the optical fiber cladding. Various types of unclad fiber sensors have been reported in the literature^25–27^. Although tapered and unclad fiber sensors offer high sensitivity, the fragility of the fiber due to its small structure presents a significant obstacle for being used in real-world applications^28^.

Partial removal of the fiber’s cladding offers distinct advantages over the previously mentioned configurations. The core can be exposed specifically at a region of interest while keeping the rest of the fiber intact, making it relatively robust^29,30^. In fiber lasers and amplifiers, breaking circular symmetry is known to enhance gain core excitation^31,32^ and can similarly be applied to evanescent field sensing. Side-polished fibers have been commonly used in interferometer-^33,34^ and coupler-based structures^35^ for sensing. Several theoretical studies have been performed to analyze mode propagation^36–38^ and loss characteristics and transmission behavior of side-polished fibers^39–41^. D-shaped optical fiber sensors have been similarly applied in refractive index sensing^42–44^, especially when coated with metallic layers to exploit the surface plasmon resonance (SPR) effect, enabling the analysis of changes in transmitted power and shifts in resonance wavelength^28,29,45–47^. Lossy mode resonance (LMR)-based D-shaped fiber sensors, which utilize nano-scale thin metal oxides, have demonstrated superior sensitivity compared to SPR-based sensors^48^. These LMR-based configurations have been successfully applied to highly sensitive refractive index sensing^48^ and label-free biomolecule detection at femtomolar concentrations, as shown in the detection of immunoglobulins (IgGs)^49^. Further enhancement in the detection limit of IgGs was achieved using a multilayer dielectric structure forming a photonic band gap on a D-shaped fiber, enabling the excitation of Bloch surface waves (BSWs) at the interface of two dielectric media^50^. Theoretical models have further been developed to investigate SPR-based D-shaped fiber sensors^28,51,52^.

In contrast, relatively fewer studies have investigated D-shaped fiber optic sensors operating based on fluorescence emission^53^. On the theoretical side, studies have delved into fluorescence capturing in optical fibers^54,55^, D-shaped fibers^56^, and microstructured optical fibers^1,2^. Marcuse^3^ investigated the excitation of fiber modes using an incoherent light source. The source was modeled as a circular disk containing dipoles with random phases and orientations, with the current density treated as a random function. In a later study^54^, the same author developed a theory for fluorescence recapturing in the guided modes of a multimode optical fiber, where incoherent fluorescence centers distributed in the cladding are excited by an external source. Henderson $\it et al$.^55^, analyzed the emission behavior of a single photon emitter, modeled as a dipole, coupled to the guided modes of a step-index optical fiber. In^56^, Henry utilized the scalar modal fields to investigate the pump absorption and fluorescence recapturing in a D-shaped optical fiber. In-fiber excitation and fluorescence capture from a homogeneous coating in a microstructured fiber were modeled and experimentally analyzed in^1,2^.

However, existing models for fluorescence-based fibers remain limited, often focusing on individual aspects such as emission recapture or coupling efficiency, while overlooking the broader excitation–emission–collection sequence. Optimizing the collection of fluorescence into the detector is crucial for many applications, particularly in biophotonics, where the number of target species for detection is limited and the fluorescence signal is often weak due to noise and background interference. Moreover, the combined effects of spatially distributed, incoherent fluorophores interacting with complex multimodal excitation and collection, along with modal interference that shapes excitation and capture efficiency, have received little theoretical attention.

In this paper, we develop a comprehensive theoretical model that addresses the limitations of previous studies and apply it to a multimode D-shaped fiber to investigate how key parameters: polishing depth, core size, and excitation mode, affect the total fluorescence output. Sect. “Theoretical modeling” introduces the theoretical framework for modeling fluorescence-based multimode fiber sensors. Sect. “Simulation” describes its implementation for a D-shaped fiber geometry. Finally, Sect. “Results and discussion” presents simulation results, organized into three subsections that examine the influence of polishing depth, core size, and excitation mode on the fluorescence power coupled into the collection segment.

## Theoretical modeling

**Fig. 1:**
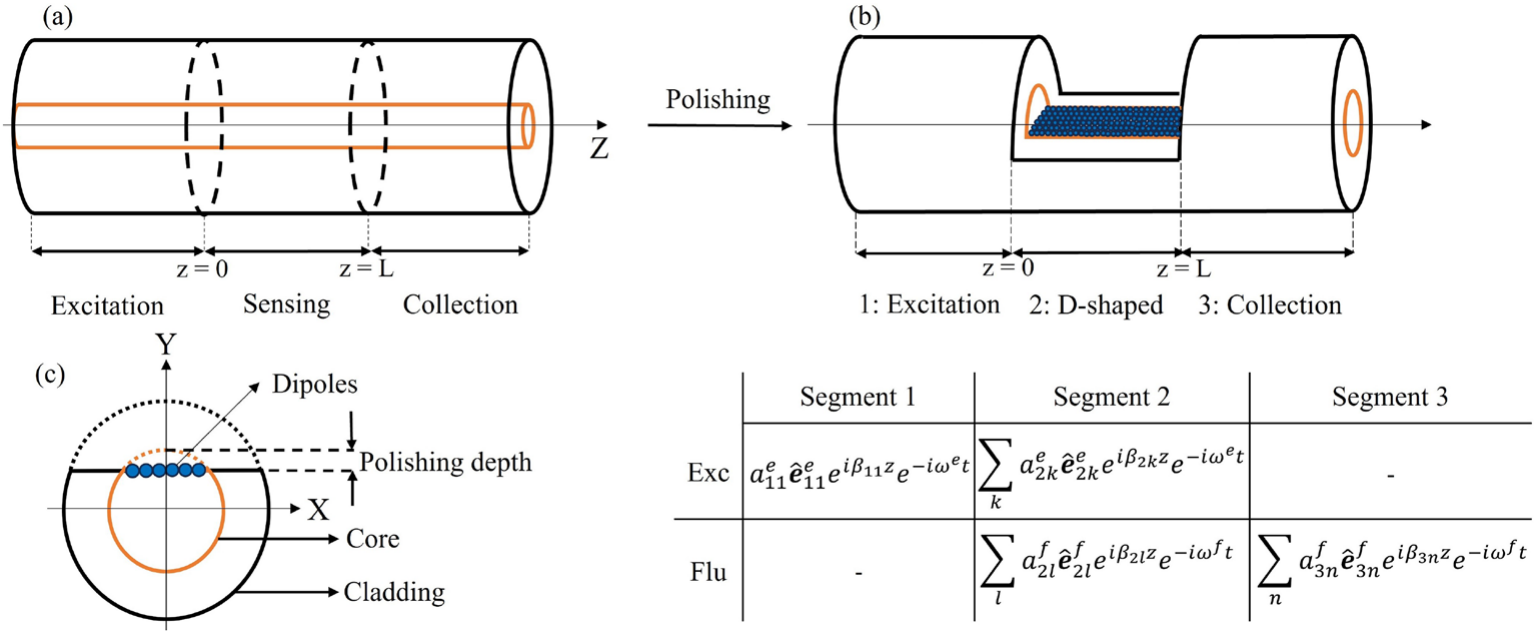
Schematic configuration of a fluorescence-based multimode optical fiber sensor, shown in (**a**) unpolished and (**b**) polished forms. The segments are labeled as follows: 1-excitation, 2-sensing/D-shaped, and 3-collection. The table illustrates the excitation (Exc) and fluorescence (Flu) fields for each segment. (**c**) Cross-sectional view of the D-shaped segment, depicting the fluorophore distribution along the *x* axis and showing polishing depth as the distance from the core’s top.

The schematic configuration of the optical fiber sensor is presented in Fig. 1a, b, before and after polishing, respectively.

The first segment is the excitation segment, where a single mode of the fiber serves as the excitation mode. This mode is initially coupled to all modes within the D-shaped second segment, exciting all the fluorophores deposited on this segment (see Fig. 1c). To accurately reflect coating processes in fluorescence-based experiments, where the concentration of the fluorophore is maintained constant, we adopt a fixed density of fluorophores at the core-external medium interface. The emitted fluorescence is then captured by all available modes in segment 2, which is subsequently coupled to all modes of segment 3 to transfer the emission to the detection system, assuming no reabsorption of the emitted fluorescence by the fluorophores.

Table 1 summarizes the superscripts and subscripts used throughout this section.

**Table 1:** Summary of superscripts and subscripts used in the theoretical model.

Symbol / index	Description
Superscripts	
*e*	Excitation field
*f*	Fluorescence field
Subscripts	
$s = 1, 2, 3$	Segment index: 1 = excitation, 2 = sensing (D-shaped), 3 = collection
*j*, $k, k^\prime$, $l, l^\prime$, *n*	Modal indices in different segments
*m*	Dipole ensemble index
*d*	Dipole index within ensemble *m*

### Excitation

The excitation process is partitioned into two consecutive processes: the first entails the in-coupling of excitation modes in segment 2 with a single mode of segment 1, while the second addresses the fluorophores excitation in segment 2. The electromagnetic fields in any segments, $s = 1, 2, 3$, of the fiber are expressed in terms of forward guided modes (excluding backward and radiation modes)^57^:

1 $$\begin{aligned} \boldsymbol{E}^{e,f}_s(x,y,z,t)= & \sum _{j} a^{e,f}_{sj}\boldsymbol{\hat{e}}^{e,f}_{sj}(x,y)\,\exp {({i\beta ^{e,f}_{sj} z})}\,\exp {({-i\omega ^{e,f} t})}, \end{aligned}$$

2 $$\begin{aligned} \boldsymbol{H}^{e,f}_s(x,y,z,t)= & \sum _{j} a^{e,f}_{sj}\boldsymbol{\hat{h}}^{e,f}_{sj}(x,y)\,\exp {({i\beta ^{e,f}_{sj} z})}\,\exp {({-i\omega ^{e,f} t})}, \end{aligned}$$

where, superscripts *e* and *f* refer to excitation and fluorescence fields, respectively, the modal amplitude and propagation constant of the $j^{th}$ forward propagating mode are represented as $a^{e,f}_{sj}$ and $\beta ^{e,f}_{sj}$, and the angular frequency is indicated as $\omega ^{e,f}$. Bold letters indicate vectors. This model considers only guided modes in each fiber segment in both excitation and emission stages. Excitation and fluorescence fields for the three segments are presented in the table in Fig. 1b. The orthonormal electric and magnetic modal fields, $\boldsymbol{\hat{e}}^{e,f}_{sj}(x,y)$ and $\boldsymbol{\hat{h}}^{e,f}_{sj}(x,y)$, can be constructed from modal fields using^57^:

3 $$\begin{aligned} \boldsymbol{\hat{e}}^{e,f}_{sj}=\frac{\boldsymbol{e}^{e,f}_{sj}}{\sqrt{N^{e,f}_{sj}}}, \hspace{0.25cm} \boldsymbol{\hat{h}}^{e,f}_{sj}=\frac{\boldsymbol{h}^{e,f}_{sj}}{\sqrt{N^{e,f}_{sj}}}, \hspace{0.25cm} \frac{1}{2}\int _{A_\infty }{\boldsymbol{\hat{e}}^{e,f}_{s,j}\times \boldsymbol{\hat{h}} ^{*e,f}_{s,k}\cdot \boldsymbol{\hat{z}}\,dA} = \delta _{jk}, \end{aligned}$$

where:

4 $$\begin{aligned} N^{e,f}_{sj}=\frac{1}{2} \int _{A_\infty }{\boldsymbol{e}^{e,f}_{sj} \times \boldsymbol{h}^{*e,f}_{sj}\cdot \boldsymbol{\hat{z}}\,dA}. \end{aligned}$$

Since the fields are orthonormal, $|a^{e,f}_{sj} |^2$ represents the power carried by each mode. The coupling of the excitation field from segment 1 to the modes in segment 2 can be quantified by the in-coupling coefficient $a^e_{2k}$, calculated by applying the continuity of the transverse electric fields at the boundary between segments 1 and 2 at $z=0$ and orthonormality condition (Eq. (3)):

5 $$\begin{aligned} {a^e_{2k}} = \frac{1}{2}a^e_{11}\int _{A_\infty } {\boldsymbol{\hat{e}}^e_{11}\times \boldsymbol{\hat{h}}^{*e}_{2k}\cdot \boldsymbol{\hat{z}}\,dA}, \end{aligned}$$

where, *k* is an excitation mode index in segment 2. The in-coupling efficiency, $CE_{in}$, can be calculated as: $CE_{in} = \frac{1}{|a^e_{11}|^2}\sum _{k}|a^e_{2k} |^2$. This study assumes negligible optical power loss due to Fresnel reflection at the interface between the segments. The highest amplitude reflection coefficient at the refractive-index boundary between the fiber ($\mathrm {n_{core}}$ = 1.45) and the external medium (n = 1.33, for full polished segment 2) is approximately 0.043 ($\approx$ 4%), which corresponds to a power reflection of only $R=|r |^2 \approx$ 0.2%. The intrinsic material loss of the optical fiber in the presented sensing configuration is negligible, as silica exhibits a loss of approximately 0.01 dB/m at 500 *nm*^58^. Additionally, loss due to surface roughness in the polished area has been ignored, as it is highly dependent on the polishing method. Various techniques, such as chemical etching^59^, $\mathrm {CO_2}$ laser polishing^60^, femtosecond laser ablation^43^, mechanical methods like V-groove polishing^39^ and wheel polishing^61^, as well as D-shaped preform development in a fiber drawing tower^62^, can influence the final surface quality and, consequently, the extent of scattering losses. Note that losses resulting from the coupling of excitation and emission signals between different segments, as well as the coupling of the fluorescence signal into the guided modes of segment 2, have inherently been accounted for in our model since the coupling coefficients in the following equations have been only calculated for the guided modes. When the modes in segment 2 are excited, the fluorophores–modeled as dipoles–are also excited. The dipoles located in segment 2 are represented by the position vector $\boldsymbol{r}_m$. At each position, there is an ensemble of dipoles. The excitation polarization vector at position $\boldsymbol{r}_m$, representing the dipole moment density at excitation wavelength, can be written as:

6 $$\begin{aligned} \boldsymbol{P}^e_{2m} = \alpha \boldsymbol{E}^e_2(\boldsymbol{r}_m)\delta (\boldsymbol{r}-\boldsymbol{r}_m), \end{aligned}$$

where, $\alpha$ is the polarizability of the molecule, and $\boldsymbol{E}^e_2(\boldsymbol{r}_m)$ is the excitation electric field at the position of the ensemble.

### Fluorescence capturing

Upon excitation, the dipole undergoes a non-radiative transition to some intermediate states, from which it emits electromagnetic radiation at the fluorescence wavelength and decays to the ground state. We associate a polarization vector with the fluorescence emission, representing the dipole moment density at the emission wavelength, and assume that it is proportional to $|\boldsymbol{P}^e_{2m}|$, i.e.,

7 $$\begin{aligned} \boldsymbol{P}^f_{2md} = \eta \boldsymbol{\hat{\rho }}_{md}\, |\boldsymbol{P}^e_{2m} |\, \exp {({-i\omega ^f(t - t_\tau )})} = \eta \boldsymbol{\hat{\rho }}_{md}\, |\boldsymbol{P}^e_{2m} |\, \exp {({-i\omega ^f t})}\, \exp {({i\Phi _{md}})}, \end{aligned}$$

where, $\eta$ is the quantum efficiency of the molecule, and $\boldsymbol{\hat{\rho }}_{md}$ is a random unit vector indicating the random orientation of the dipoles in the ensemble. Here, $t_\tau$ is determined by the dipole’s lifetime decay and is a random number, making $\Phi _{md} = \omega ^ft_\tau$, a uniform random number between 0 and $2\pi$. The current density associated with the time derivative of the fluorescence polarization vector can be expressed as:

8 $$\begin{aligned} \boldsymbol{J}^f_{2md} = -i\omega ^f\eta \boldsymbol{\hat{\rho }}_{md}\,|\boldsymbol{P}^e_{2m}|\,\exp {({-i\omega ^ft})}\,\exp {({i\Phi _{md}})}. \end{aligned}$$

The expression for modal amplitude $a^f_{2lmd}$ of the $l^{th}$ fluorescence-capturing mode in segment 2, when excited with $\boldsymbol{J}^f_{2md}$, can be derived from the formulation presented in^57^:

9 $$\begin{aligned} a^f_{2lmd}=\frac{-1}{4}\int _{V} \boldsymbol{\hat{e}}^{*f}_{2l}(\boldsymbol{r})\cdot \boldsymbol{J}^f_{2md}(\boldsymbol{r}) \,\exp {({-i\beta ^f_{2l}z})}\,dV, \end{aligned}$$

where, *V* is the volume along segment 2 where the dipoles are positioned. The emission of a dipole into different modes of an optical fiber is a stochastic process, meaning that there is a certain probability for the decay of dipole to the ground state via different modes. These probabilities depend on fiber modes, and thus, we introduce a subscript *l* to the phase, $\tilde{\Phi }_{lmd}$, to represent this random process. Hence, using Eq. (8), Eq. (9) can be written as:

10 $$\begin{aligned} a^f_{2lmd} = \frac{i\omega ^f\eta }{4}\,\boldsymbol{\hat{e}}^{*f}_{2l}(\boldsymbol{r}_m)\cdot \boldsymbol{\hat{\rho }}_{md}\,|\boldsymbol{P}^e_{2m}|\,\exp {({i\tilde{\Phi }_{lmd}})}\,\exp {({-i\beta ^f_{2l}z_m})}. \end{aligned}$$

Our model uses a uniform dipole density to isolate the optical effects of excitation, fluorescence generation, and mode coupling. If the fluorophore coating were spatially non-uniform, the fluorescence would be scaled by the local dipole density. In this case, $a_{2lmd}^f$ which already depends on the dipole position $\boldsymbol{r}_m=(x_m,y_m,z_m)$, becomes a spatially varying quantity.

Summing up all modes, the fluorescence power captured in segment 2 from the ensemble at position $\boldsymbol{r}_m$ is given by $\sum _l|a^f_{2lmd}|^2$. In the isotropic case considered in this study, by substituting $\boldsymbol{\hat{\rho }}_{md}$ as a unit vector defined as $\boldsymbol{\hat{\rho }}_{md} = \sin {\theta }\cos {\phi } \boldsymbol{\hat{x}} + \sin {\theta }\sin {\phi } \boldsymbol{\hat{y}} + \cos {\theta } \boldsymbol{\hat{z}}$ and integrating solid angle $d\Omega = \sin {\theta }d\theta d\phi$ over the surface of a unit sphere, we obtain the power captured in segment 2 from the ensemble at position $\boldsymbol{r}_m$ as:

11 $$\begin{aligned} P^f_{2m} = \frac{(\omega ^f)^2\eta ^2\pi }{12}\sum _l|\boldsymbol{P}^e_{2m}|^2|\boldsymbol{\hat{e}}^{*f}_{2l}(\boldsymbol{r}_m)|^2. \end{aligned}$$

For fluorophores with anisotropic or partially oriented dipole distributions, the solid-angle integral above is replaced by an angular weighting function representing the dipole orientation statistics. Using Eq. (1), (6), and (11), and summing over all the ensembles at different locations $\boldsymbol{r}_m$, the total captured fluorescence power can be expressed as:

12 $$\begin{aligned} P^f_2 = \frac{(\omega ^f)^2\eta ^2\alpha ^2\pi }{12}\sum _m\sum _l\sum _k\sum _{k^{\prime }}[a^e_{2k} a^{*e}_{2k^{\prime }}\boldsymbol{\hat{e}}^e_{2k}(\boldsymbol{r}_m)\cdot \boldsymbol{\hat{e}}^{*e}_{2k^{\prime }}(\boldsymbol{r}_m)\,\exp {({i(\beta ^e_{2k}-\beta ^e_{2k^{\prime }})z_m})}]\,|\boldsymbol{\hat{e}}^{*f}_{2l}(\boldsymbol{r}_m)|^2. \end{aligned}$$

Eq. (12), when expanded, contains terms where $k=k^{\prime }$, which results in the following expression for $P^f_2$ as:

13 $$\begin{aligned} P^f_2 = \frac{(\omega ^f)^2\eta ^2\alpha ^2\pi }{12}\sum _m\sum _l\sum _k|a^e_{2k}|^2|\boldsymbol{\hat{e}}^{e}_{2k}(\boldsymbol{r}_m)|^2|\boldsymbol{\hat{e}}^{*f}_{2l}(\boldsymbol{r}_m)|^2. \end{aligned}$$

The cross terms in Eq. (12), i.e, $k\ne k^\prime$, include the phase factor $\exp {({i(\beta ^e_{2k}-\beta ^e_{2k^{\prime }})z_m})}$. The oscillatory nature of the phase factor causes the cross terms to average out to zero when summing over all the dipoles positioned along the length of segment 2 for practical length scales (>3 cm). We demonstrate the validity of this approximation in Sect. “Fluorescence behavior in a D-shaped fiber”. Therefore, Eq. (13) represents the total captured fluorescence power.

We emphasize that the above formulations remain valid for the special case of a single dipole located at position $\boldsymbol{r}_d$ with a fixed orientation $\boldsymbol{\hat{\rho }}_d$. In this scenario, the spherical integration is no longer required in Eq. (11). Consequently, in Eqs. (11), (12), and (13), the term $|\boldsymbol{\hat{e}}^{*f}_{2l}(\boldsymbol{r}_m)|^2$ is replaced by $|\boldsymbol{\hat{e}}^{*f}_{2l}(\boldsymbol{r}_d)\cdot \boldsymbol{\hat{\rho }}_d|^2$. Furthermore, there is no summation over the index *m* in Eqs. (12) and (13). The corresponding coefficients in Eq. (11) and Eqs. (12) and (13) are modified to $\frac{(\omega ^f)^2\eta ^2}{16}$ and $\frac{(\omega ^f)^2\eta ^2\alpha ^2}{16}$, respectively.

### Fluorescence coupling

To quantify the coupling of fluorescence into the $n^{th}$ mode of segment 3, the modal amplitude, $a^f_{3nmd}$, is calculated by applying the continuity condition of the transverse electric field component at the boundary between segments 2 and 3 at $z = L$ and orthonormality condition (Eq. (3)):

14 $$\begin{aligned} a^f_{3nmd} = \frac{1}{2}\sum _l a^f_{2lmd} \,\exp {({i\beta ^f_{2l}L})}\int _{A_\infty }\boldsymbol{\hat{e}}^f_{2l}\times \boldsymbol{\hat{h}}^{*f}_{3n}\cdot \boldsymbol{\hat{z}}\,dA. \end{aligned}$$

By defining $W_{ln}= \int _{A_\infty }\boldsymbol{\hat{e}}^f_{2l}\times \boldsymbol{\hat{h}}^{*f}_{3n}\cdot \boldsymbol{\hat{z}}\,dA$, and substituting Eq. (10) in Eq. (14), $a^f_{3nmd}$ can be written as:

15 $$\begin{aligned} a^f_{3nmd} = \frac{i\omega ^f\eta }{8}\sum _l \boldsymbol{\hat{e}}^{*f}_{2l}(\boldsymbol{r}_m)\cdot \boldsymbol{\hat{\rho }}_{md}\,|\boldsymbol{P}^e_{2m}|\,\exp {({i\tilde{\Phi }_{lmd}})}\,\exp {({i\beta ^f_{2l}L})} \,\exp {({-i\beta ^f_{2l}z_m})}\, W_{ln}. \end{aligned}$$

Using Eq. (15), the fluorescence power coupled to modes of segment 3 from the ensemble at position $\boldsymbol{r}_m$, can be calculated as $P^f_{3md}=\sum _n|a^f_{3nmd}|^2$:

16 $$\begin{aligned} P^f_{3md}&= \frac{(\omega ^f)^2\eta ^2}{64} \sum _n \sum _l \sum _{l^\prime } \Big ( \boldsymbol{\hat{e}}^{*f}_{2l}(\boldsymbol{r}_m) \cdot \boldsymbol{\hat{\rho }}_{md}\, |\boldsymbol{P}^e_{2m} |\,\exp {({i(\tilde{\Phi }_{lmd} + \beta ^f_{2l} L - \beta ^f_{2l} z_m)})}\, W_{ln} \Big ) \nonumber \\&\times \Big ( \boldsymbol{\hat{e}}^{f}_{2l^\prime }(\boldsymbol{r}_m) \cdot \boldsymbol{\hat{\rho }}_{md}\, |\boldsymbol{P}^{e}_{2m} |\,\exp {({-i(\tilde{\Phi }_{l^\prime md} + \beta ^f_{2l^\prime } L - \beta ^f_{2l^\prime } z_m)})}\, W^*_{l^\prime n} \Big ) \end{aligned}$$

By integrating over all possible orientations of the dipoles in an ensemble, the power is then given by:

17 $$\begin{aligned} P^f_{3m}&= \frac{(\omega ^f)^2\eta ^2}{64}\int d\Omega \sum _n\sum _l\sum _{l^\prime } \Big (\boldsymbol{\hat{e}}^{*f}_{2l}(\boldsymbol{r}_m)\cdot \boldsymbol{\hat{\rho }}_{md}\,|\boldsymbol{P}^e_{2m}|\,\exp {({i(\tilde{\Phi }_{lmd}+\beta ^f_{2l}L-\beta ^f_{2l}z_m)})}\,W_{ln} \Big ) \nonumber \\&\times \Big (\boldsymbol{\hat{e}}^{f}_{2l^\prime }(\boldsymbol{r}_m)\cdot \boldsymbol{\hat{\rho }}_{md}\,|\boldsymbol{P}^e_{2m}|\,\exp {({-i(\tilde{\Phi }_{l^\prime md}+\beta ^f_{2l^\prime }L-\beta ^f_{2l^\prime }z_m)})}\, W^*_{l^\prime n}\Big ). \end{aligned}$$

Subsequently, the total fluorescence power coupled to segment 3, can be defined by summing over all positions of the ensembles along the fiber axis as, $P^f_3 = \sum _m P^f_{3m}$. The expression contains non-cross terms, where $l=l^\prime$, and leads to the following expression for $P^f_3$:

18 $$\begin{aligned} P^f_3 = \frac{(\omega ^f)^2\eta ^2\alpha ^2\pi }{48}\sum _m\sum _n\sum _l\sum _k|a^e_{2k}|^2|W_{ln}|^2|\boldsymbol{\hat{e}}^e_{2k}(\boldsymbol{r}_m)|^2|\boldsymbol{\hat{e}}^{*f}_{2l}(\boldsymbol{r}_m)|^2. \end{aligned}$$

There are also terms corresponding to the interfering cross terms, where $l\ne l^\prime$. At each dipole position, the phases $\tilde{\Phi }_{lmd}$ and $\tilde{\Phi }_{l^\prime md}$ are random, leading to a random phase factor in this term. Upon averaging over all dipole positions, these random phases will cancel out, resulting in no net contribution from the cross terms. Eq. (18) indicates that in order to calculate the total coupled power, we first need to calculate the power of an individual dipole, when excited by one mode, emits into one mode, and the emission is coupled to one mode, then summing over the combination of all dipoles, excitation, capturing, and collection modes. The out-coupling efficiency, $CE_{out}$, can be then defined as the ratio of the coupled fluorescence power to segment 3 and the captured fluorescence power in segment 2:

19 $$\begin{aligned} CE_{out} = \frac{P^f_{3}}{P^f_{2}}. \end{aligned}$$

## Simulation

To demonstrate the application of the theoretical framework developed in Sect. “Theoretical modeling”, we apply it to a D-shaped fiber optic sensor as a representative case study. The objective is to determine the optimum conditions–fiber core size, polishing depth, and excitation mode–for maximizing fluorescence output. The fiber was modeled in COMSOL Multiphysics using the wave optics module and the electromagnetic wave frequency domain solver to compute the modal electric and magnetic fields. These fields were then extracted and used to calculate the total fluorescence power.

**Table 2:** Simulation parameters used for modeling.

Parameter	Value
Wavelength	530 nm
Core diameters	4, 7, 10, 14, 21 $\mu$m
Cladding diameter	Twice the core diameter
Refractive index (core)	1.45
Refractive index (cladding)	1.44
Refractive index (external medium)	1.33
PML thickness	1060 nm
Polishing depth resolution	100 nm

The key simulation parameters are listed in Table 2. Assuming that the excitation and fluorescence wavelengths are close to each other, we evaluated all the fields for a single wavelength. Perfectly matched layer (PML) and scattering boundary conditions were applied. The polishing depth was defined from the core’s top (see Fig. 1c), with the refractive index of the polished area matching the external medium. The simulation region was divided into domains with varying mesh sizes to ensure the convergence of the results. The fiber core diameters used in this study represent practical and commercially available optical fibers to illustrate the concept, assuming that larger core diameters would follow the same trend. The cladding diameter for each fiber was set to twice the core diameter solely to constrain the computational domain. A larger cladding diameter would not affect the results, as the electromagnetic field does not extend significantly into the cladding.

The electric and magnetic fields were processed in MATLAB. To interpolate these fields across the core and cladding domains, we defined a mesh grid with a resolution of 150 *nm* for a 14 $\mu m$-core fiber, with a scaling factor applied for other core diameters. At the core–external medium boundary, the displacement field $\varepsilon _r \boldsymbol{E}$ was interpolated to account for discontinuities in the normal electric field component. The increment of polishing depth was set to 100 *nm*, starting from 100 *nm* below the top of the core to where FEM could no longer find bounded modes in the core. This step size was selected to ensure subwavelength resolution and was sufficient to limit the change in the number of supported modes, including degeneracy, to no more than two between successive depths. Furthermore, we did not observe any significant change in the general behavior of fluorescence collection when finer resolutions were used. For this study, a fixed density of 20 dipole positions per micrometer was used at the core-external medium interface.

To address the incoherent nature of excitation and fluorescence emission, we analyzed each dipole individually to eliminate interference effects. We assumed that the excitation mode in segment 1 is coupled to all modes in segment 2. One mode in segment 2 excites a dipole, and the dipole’s emission is captured by a mode in segment 2. This captured fluorescence is then coupled to a mode in segment 3. We calculated the power output for this configuration. This process was repeated systematically for all excitation modes, dipoles, capturing modes in segment 2, and coupling modes in segment 3, allowing us to sum the power contributions from each configuration and obtain the total emission.

## Results and discussion

The excitation field in each segment is governed by the available modes and their amplitudes, determined via overlap integrals between the modes of adjacent segments. Quite differently, the fluorescence field depends on the total excitation electric field magnitude and the modal fluorescence electric fields at the dipole position (as shown in Eq. 11).

In an unpolished fiber (a 100 *nm* polished segment 2), the excitation mode distributions in segments 1 and 2, and the fluorescence mode distributions in segments 2 and 3, remain the same due to minimal structural change. With deeper polishing, these distributions differ, as each segment’s modal content and coupling coefficients–defined by overlap integrals–change accordingly.

We first investigate the fluorescence capture behavior of a 14 $\mu m$-core fiber when excited with the fundamental mode (FM) of segment 1. We then analyze the impact of varying core size on fluorescence output and optimal polishing depth. Finally, we examine the influence of excitation modes on these parameters. To facilitate comparison across different core diameters and excitation modes, all fluorescence powers have been normalized to the output power of the 100 *nm* polish of the 14 $\mu m$ core diameter when excited with the FM. This reference value is set to 1 $\mu W$, which aligns with typical experimental results^17^. To calculate fluorescence power in this study using the equations provided in Sect. “Theoretical modeling”, we employed Rhodamine B as the fluorophore, with $\alpha = 5.71 \times 10^{-39} \,\mathrm {C\cdot m^2/V}$^63^, and set $\eta =1$.

### Fluorescence behavior in a D-shaped fiber

Before analyzing the fluorescence power captured in segment 2, it is important to validate the averaging approximation used to derive Eq. 13 from Eq. 12. The assumption is that the oscillatory cross terms in Eq. 12 vanish due to phase averaging over propagation lengths relevant to practical sensor designs.

**Fig. 2:**
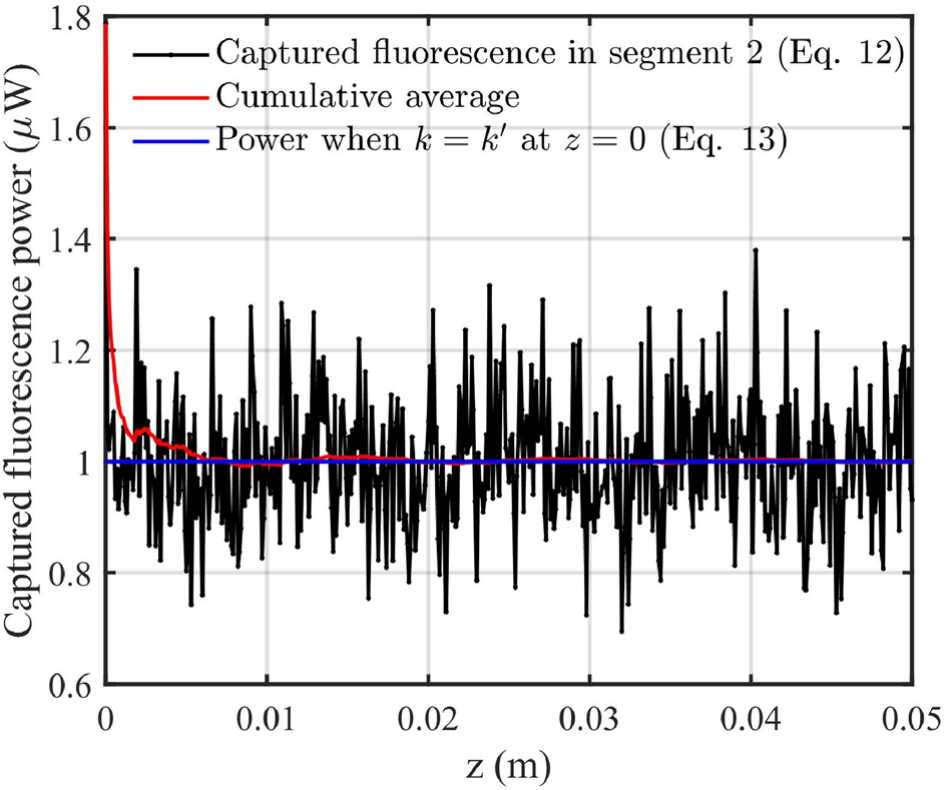
Captured fluorescence power calculated using Eq. (12) (black), cumulative average using Eq. (12) (red), and predicted value from Eq. (13) (blue), for a 14 $\mu m$ core diameter fiber with 100 *nm* polishing depth over the polishing length from 0 to 5 *cm*.

To test this, simulations were performed for all core diameters over a 0–5 *cm* propagation range, considering three polishing depths: 100 *nm*, mid-core, and the final depth. Figure 2 shows a representative case for a 14 $\mu m$-core fiber at 100 *nm* polishing depth. The cumulative average of captured fluorescence (red curve) stabilizes to within 1% of the value predicted by Eq. 13 (blue line) after approximately 6 *mm* of propagation. Similar convergence behavior was observed across all simulated cases, with averaging lengths ranging from 0 to 3 *cm*.

With this validation in place, we next analyze the fluorescence power captured in segment 2. It is important to examine how the number of dipoles and modes, the evanescent field at the dipoles’ position, and the in-coupling of the excitation mode from segment 1 to 2 behave as a function of polishing depth.

**Fig. 3:**
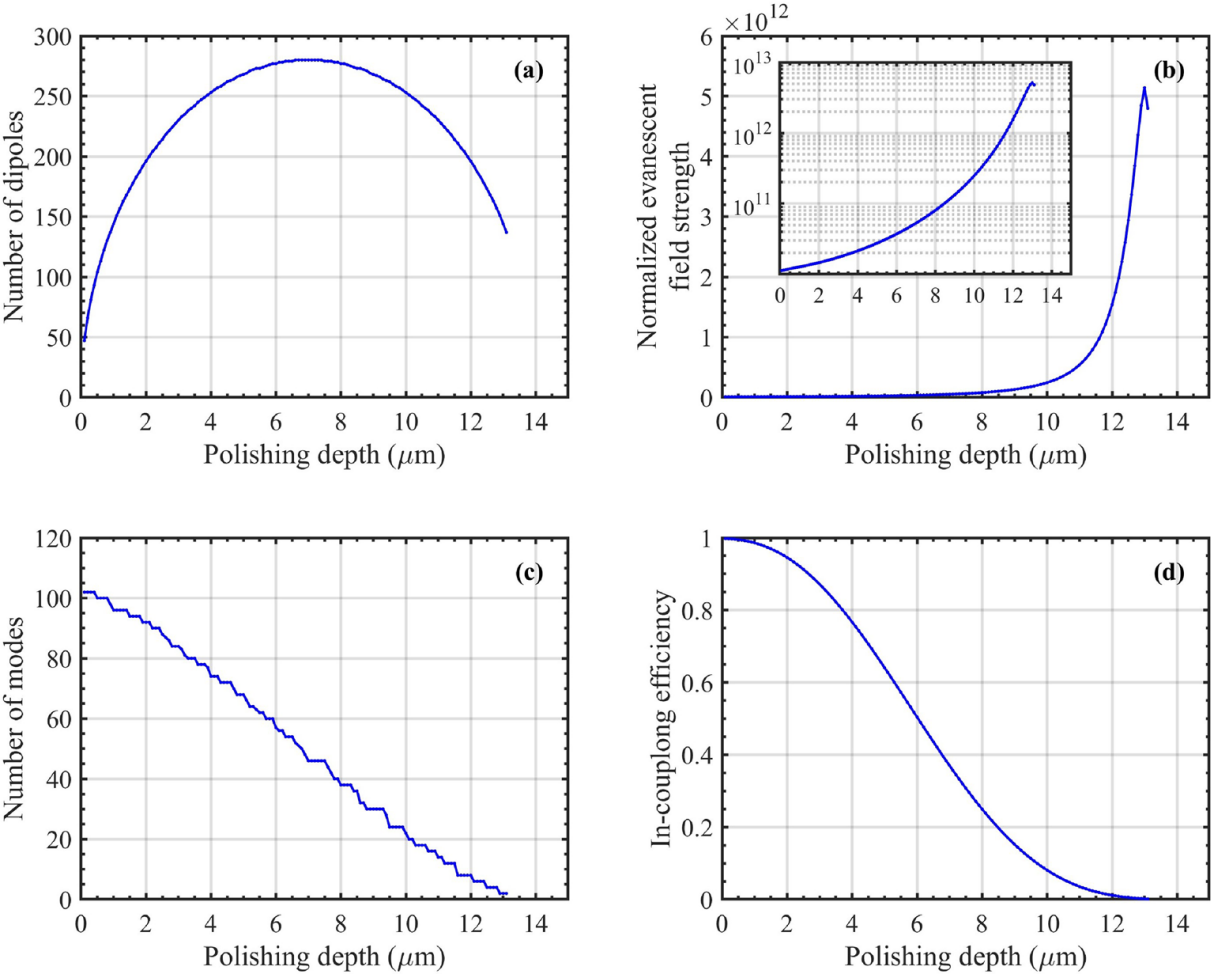
(**a**) Number of dipoles, (**b**) evanescent field strength with a log-scale inset for the fundamental mode, (**c**) number of modes, and (**d**) in-coupling efficiency, as functions of polishing depth for a 14 $\mu m$-core diameter fiber.

Fig. 3 illustrates these effects for a multimode fiber with a 14 $\mu m$ core diameter, serving as an example when excited with the FM. As the polishing depth increases, the number of dipoles initially increases toward the middle of the core and then decreases with further polishing, as illustrated in Fig. 3a. Assuming a constant dipole density, polishing toward the middle of the core expands the region where dipoles are present, followed by a reduction in this area as polishing continues. The evanescent field in Fig. 3b was calculated as the total strength of the electric field at the dipole positions, $\sum _m |\boldsymbol{\hat{e}}_{2}(\boldsymbol{r}_m)|^2$, normalized by the total number of dipoles at each polishing depth. Initially, as the core is progressively polished, the evanescent field at the surface increases due to reduced mode confinement. However, beyond a certain polishing depth (13 $\mu m$ in Fig. 3b), the field strength at the surface decreases as the core becomes too small to sustain tight confinement, causing the mode to expand further from the surface and reducing the field strength. This is similar to the behavior observed for the mode area of optical nanowires^64^. Meanwhile, as the core is polished, the number of modes in segment 2 reduces, as shown in Fig. 3c, which is attributed to the decrease in the core’s cross-sectional area. A smaller core means a lower numerical aperture (NA) and a higher cut-off condition for higher-order modes (HoMs), causing them to no longer be guided. The in-coupling efficiency from segment 1 to segment 2 continuously decreases with increasing polishing depth due to smaller number of modes and increased mismatch between mode profiles in the segment.

**Fig. 4:**
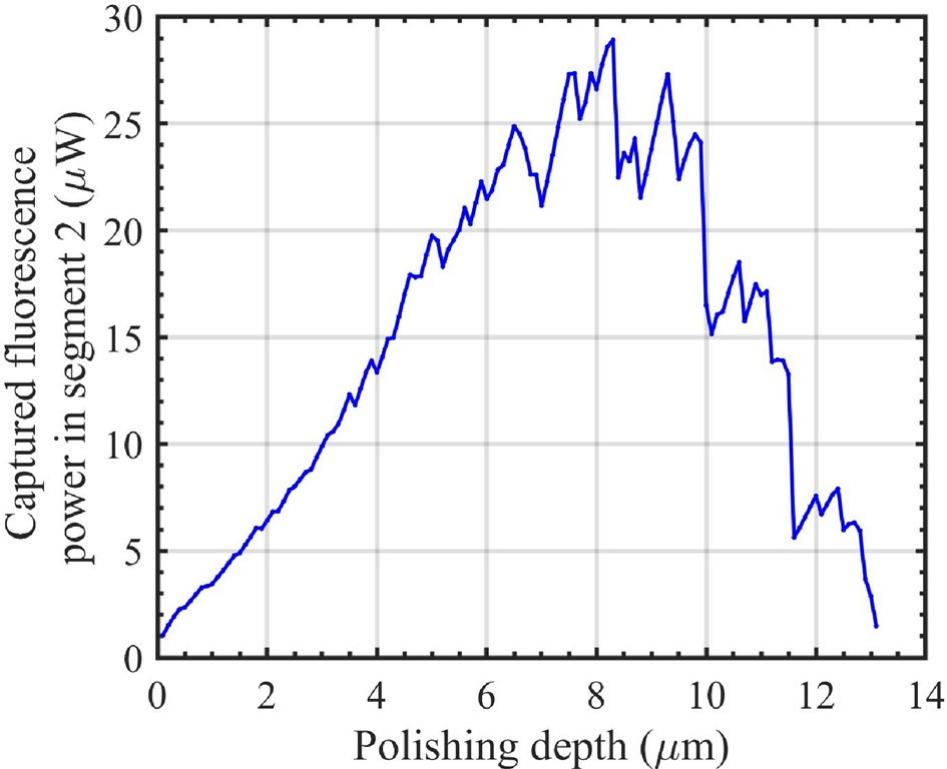
Fluorescence power captured in segment 2 of a 14 $\mu m$-core fiber as a function of polishing depth. The captured fluorescence increases with polishing depth up to approximately the middle of the core, after which it decreases, reaching a minimum near the bottom of the core.

The overall increase in dipole number and the evanescent field with polishing towards the middle of the core outweighs the reduction in number of modes and excitation in-coupling, leading to a net increase in fluorescence captured across all available modes in segment 2 (calculated using Eq. (13)), as shown in Fig. 4 for a 14-$\mu m$ core fiber.

As the polishing depth progresses further towards the bottom of the core, the number of dipoles, modes, and the coupling of excitation into segment 2 decrease, as observed in Fig. 3a, c, d, respectively.

Therefore, despite the continued increase in the evanescent field strength, the fluorescence captured in segment 2 begins to decrease, as depicted in Fig. 4. The jagged appearance of the captured fluorescence indicates the effect of mode cut-off as the polishing increases, as shown in Fig. 3c. For polishing depths with the same number of modes available, the sharp peaks in the fluorescence curve are primarily due to the enhancement of the evanescent field before the modes are cut off^54^.

**Fig. 5:**
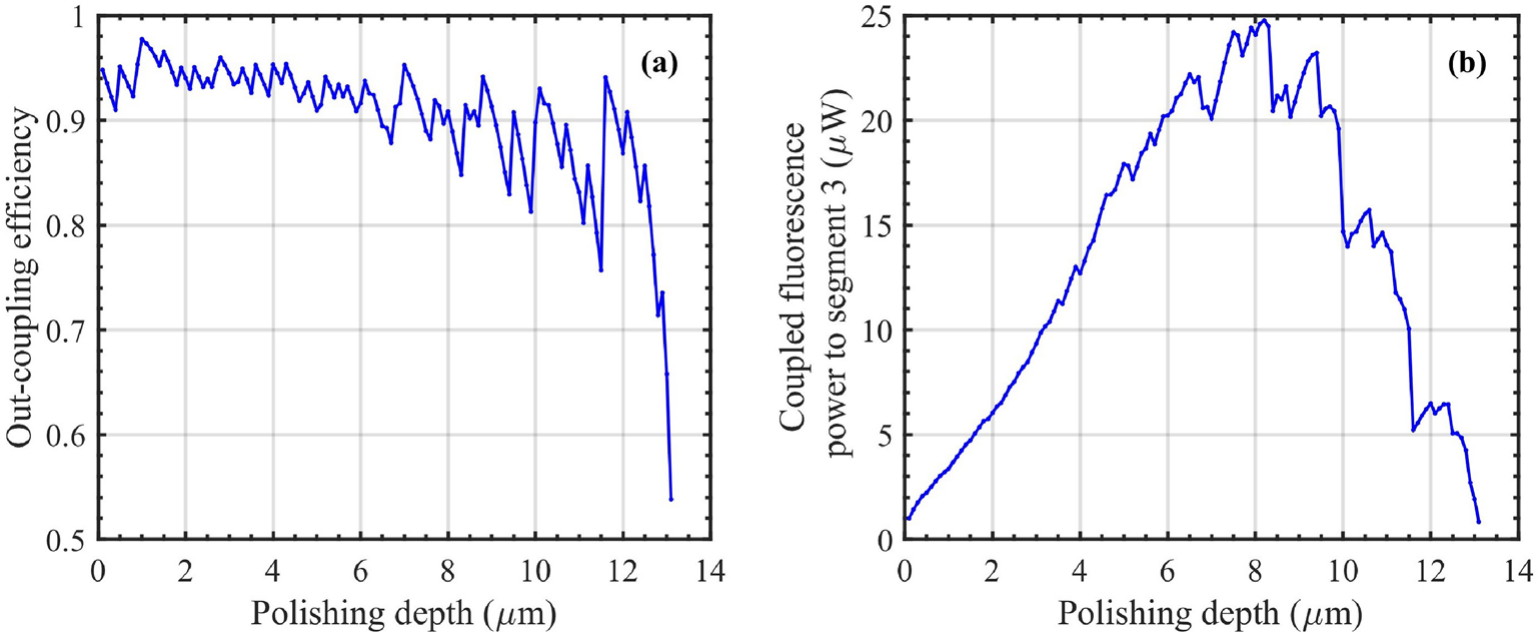
(**a**) out-coupling efficiency and (**b**) coupled fluorescence power to the segment 3 of a 14 $\mu m$-core fiber. The out-coupling efficiency to segment 3 exhibits a decreasing trend with increasing polishing depth. Similar to captured fluorescence power, the fluorescence coupled back to the fiber reaches its maximum near the middle of the fiber at 8.2 $\mu m$.

Following this, we calculated the out-coupling efficiency from segment 2 to segment 3 using Eq. (19). As shown in Fig. 5a, the out-coupling efficiency decreases with increasing polishing depth, particularly at depths nearing the core’s bottom. This reduction is due to the diminishing overlap between the evanescent field and the modes in segment 3 as the polishing progresses. Both in-coupling and out-coupling efficiencies as function of polishing depth for all core diameters are illustrated in Supplementary Fig. S1 online. In-coupling ($CE_{in} = \frac{1}{|a^e_{11}|^2}\sum _{k}|a^e_{2k} |^2$) and out-coupling efficiency (Eq. 19) are defined through overlap integrals at their respective interfaces (segments 1$\xrightarrow {}$2 and 2$\xrightarrow {}$3) for two different processes of excitation and fluorescence emission, respectively. Hence, there is no correlation between these two parameters. Considering this out-coupling efficiency, the coupled fluorescence power to segment 3, calculated using Eq. (18), exhibits a trend similar to the captured fluorescence as a function of polishing depth, as illustrated in Fig. 5b. However, the total coupled power at each depth is lower than the corresponding captured fluorescence, reflecting additional consideration of out-coupling efficiency from segment 2 to segment 3.

Based on the results presented, polishing 8.2 $\mu m$ of the core of a 14 $\mu m$ core diameter multimode optical fiber yields the maximum fluorescence power, which is nearly 25 times higher than that of fiber with the same core size polished to the core-cladding boundary.

### Fiber core size impact on coupled fluorescence power

To investigate the impact of core size on total fluorescence output when excited with the FM, simulations were conducted for four more core diameters; 4, 7, 10 and 21 $\mu m$. The analysis of the results across these core diameters reveals a consistent trend in the fluorescence power coupled back into the fiber as a function of polishing depth, similar to that observed for the 14 $\mu m$-core diameter, as discussed in Sect. “Fluorescence behavior in a D-shaped fiber”. In Table 3, the maximum fluorescence power and the optimal polishing depth–defined as the depth at which maximum fluorescence power is achieved–are presented for different core diameters, along with the optimal polishing depth expressed as a percentage of the core diameter in the last column.

The results indicate that smaller core diameters exhibit higher fluorescence power, primarily due to the stronger evanescent field present in these smaller core diameters, despite supporting fewer modes and dipoles compared to fibers with larger core sizes.

**Table 3:** Maximum fluorescence power and optimal polishing depth for various core diameters.

Core diameter ($\mu$m)	Maximum fluorescence power ($\mu$W)	Optimal polishing depth ($\mu$m)	Optimal polishing depth (%)
4	282.44	2.2	55
7	89.99	3.6	51
10	45.42	5.2	52
14	24.77	8.2	58
21	11.34	13.1	62

The analysis of the optimal polishing depth reveals an almost linear trend: smaller core diameters achieve optimal fluorescence coupling with polishing at the core’s midpoint, while larger core diameters require polishing beyond the halfway point as shown in the last column of Table 3. This is because, in smaller cores, the evanescent field extends relatively further into the cladding, making it easier to access with shallower polishing. In contrast, larger core diameters have a proportionally smaller evanescent field relative to the core size, requiring deeper polishing to effectively interact with and couple the fluorescence. The data presented in Table 3 is visually represented by the blue columns in Fig. 7.

### Excitation mode impact on coupled fluorescence power

In the results presented in Sects. “Fluorescence behavior in a D-shaped fiber” and “Fiber core size impact on coupled fluorescence power”, the excitation of segment 2 modes was conducted using the FM of segment 1. To investigate the influence of excitation mode on fluorescence output, we now excite the modes within segment 2 using individual HoMs from segment 1. Several established techniques can be employed to excite HoMs in optical fibers, including using spatial light modulator (SLM)^65^, long-period grating (LPG)^66^, and tilted light launching^67^.

**Fig. 6:**
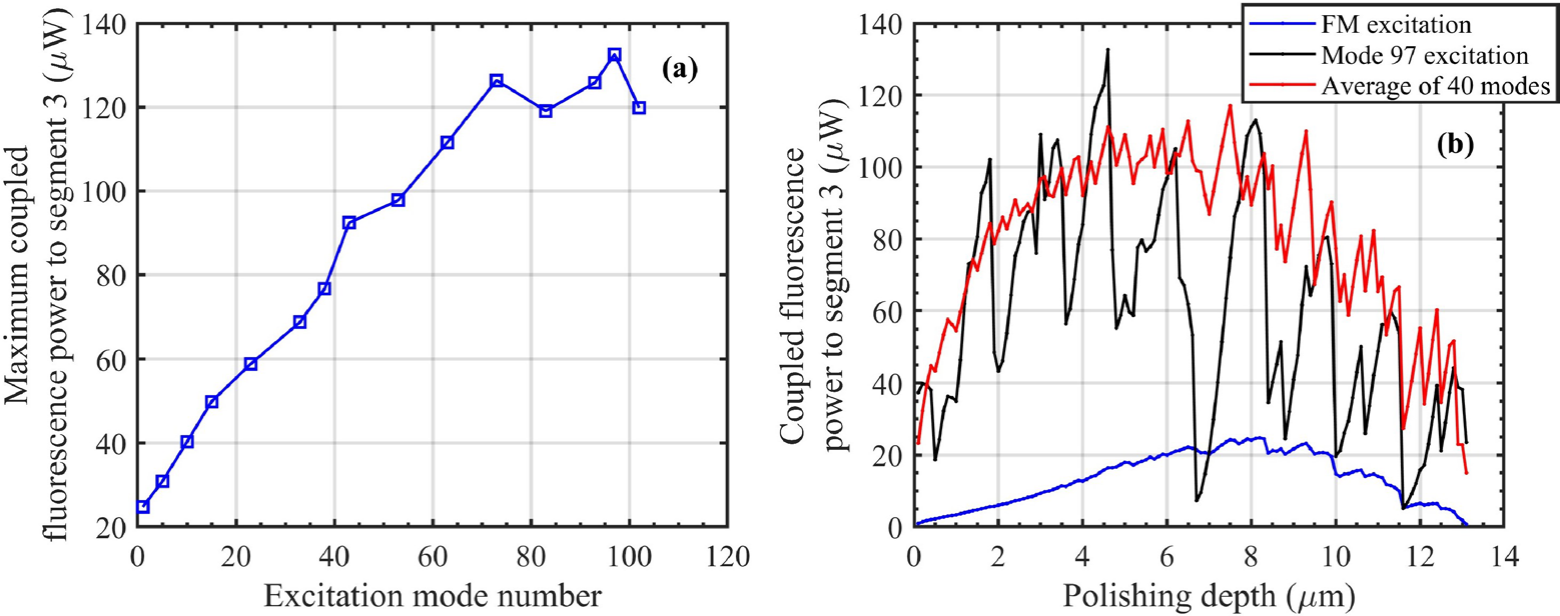
(**a**) Maximum coupled fluorescence power for various excitation modes of a 14 $\mu m$ core fiber. HoM excitation increases fluorescence output compared to FM excitation. (**b**) Coupled fluorescence power as a function of polishing depth for FM excitation (blue), HoM number 97 excitation (black), and the average of last 40 HoMs (red). HoM excitation results in greater coupled fluorescence power and a shift to shallower polishing depths.

The results indicate that excitation with HoMs leads to a stronger fluorescence output compared to FM excitation, primarily due to the higher evanescent fields associated with these modes. Fig. 6a illustrates the maximum coupled fluorescence power for a 14 $\mu m$ fiber as a function of excitation mode. Here, mode 1 represents the FM, while mode 102 denotes the highest order guided mode supported by the fiber. Fluorescence power is calculated for various excitation modes within this range, as shown in Fig. 6a. Notably, excitation with mode 97, for example, yields a fluorescence output five times greater than that achieved with the FM. The 100 *nm* resolution of the polishing depth introduces some uncertainty in pinpointing the exact mode number that maximizes coupled fluorescence in segment 3. Despite the enhanced fluorescence output provided by HoMs, this output is highly sensitive to polishing depth. As shown in Fig. 6b, the black curve (mode 97 excitation) exhibits strong variations in power with depth compared to the more stable blue curve for FM excitation. We hypothesize that the coupling to HoMs in segment 2 creates more fluctuations when excited with HoMs. This is due to the fact that HoMs in segment 2 have different cut-offs at varying polishing depths, and the behavior of their evanescent fields as a function of polishing depth are also highly complex. Furthermore, Fig. 6b demonstrates that excitation with HoMs results in maximum fluorescence power at shallower optimal polishing depths when compared to FM excitation. In practice, achieving the exact polishing depth, maintaining uniform polishing across segment 2, and controlling the number of modes excited in segment 1 can only be done within a certain tolerance. However, on average, excitation with HoMs provides a stronger fluorescence signal at a shallower depth, as shown by the red curve in Fig. 6b (average of the last 40 HoMs).

**Fig. 7:**
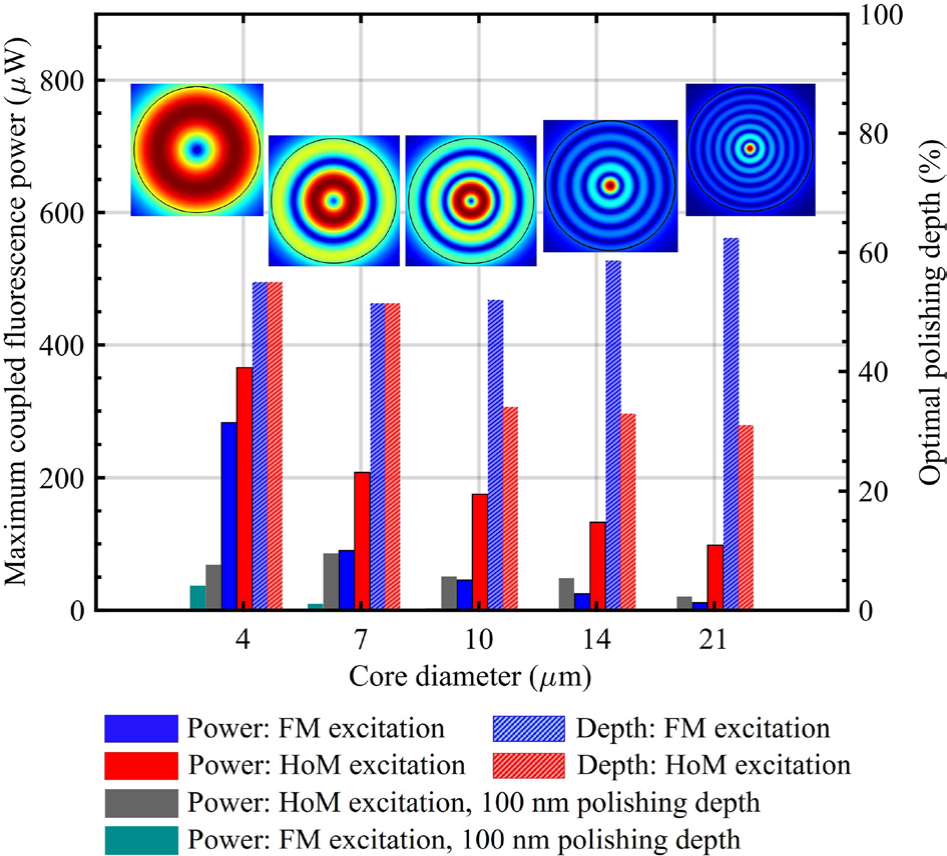
Comparison of maximum fluorescence power in segment 3 (solid columns) and optimal polishing depth of the core (stripped columns) between FM (blue and dark green) and HoM (red and gray) excitation for various core diameters. The excitation modes resulting in the maximum fluorescence output for the optimally polished fibers are indicated above each corresponding core diameter. The gray and dark green columns represent the maximum coupled fluorescence power for HoM excitation and the coupled fluorescence power for FM excitation of unpolished fibers (100 *nm* polishing).

The increase in fluorescence output with HoM excitation is consistent across various core diameters studied; 4, 7, 10, 14 and 21 $\mu m$. Fig. 7 demonstrates that HoM excitation (red solid columns)– modes 5, 19, 45, 97, and 205 for the 4, 7, 10, 14, and 21 $\mu m$ core diameters, respectively, which leads to the highest fluorescence capture– yields greater fluorescence output compared to FM excitation (blue solid columns) for all core diameters, with the difference being more pronounced for larger core diameters. The results suggest that smaller core fibers can achieve high fluorescence power, though their limited core diameter may be impractical for applications. Alternatively, larger core fibers are easier to handle and, when polished and excited with a HoM, can achieve greater fluorescence output. HoM excitation can also compensate for the reduced sensitivity typically associated with larger core fibers, making them a more practical and effective choice over smaller core fibers with FM excitation.

In Fig. 7, the dark green and gray bars represent the coupled fluorescence power for the unpolished fiber (100 *nm* polishing depth, as a reference), corresponding to FM excitation and the maximum achieved under HoM excitation, respectively. Comparison with the polished fibers reveals that: (i) the coupled fluorescence power is extremely weak under FM excitation in the unpolished case, as seen by the nearly invisible dark green bars for 10, 14, and 21 $\mu m$ core diameters; and (ii) for larger cores (14 and 21 $\mu m$), HoM excitation in the unpolished fiber leads to higher fluorescence than FM excitation in the optimally polished fibers.

The distribution of the amplitude of the electric field, representing the excitation modes that result in maximum fluorescence power, within the core for different core sizes is illustrated in Fig. 7. The relationship between optimal polishing depth and excitation with HoMs varies with fiber core diameter as depicted in Fig. 7 (stripped columns). Larger cores (10, 14, and 21 $\mu m$) exhibit shallower optimal polishing depths for achieving maximum fluorescence with HoM excitation. In contrast, this difference is not observed in smaller core fibers (4 and 7 $\mu m$), likely due to the uncertainty introduced by the 100 *nm* resolution of the polishing depth, which makes it difficult to precisely determine the optimal polishing depth for each core size. It is important to note that the simulations in this study are based on idealized modeling assumptions designed to isolate the influence of fiber geometry, polishing depth, and excitation mode on fluorescence output. In practical implementations, parameters such as polishing depth accuracy and surface roughness –both dependent on the fabrication method–and background interference (e.g., noise and autofluorescence), which is application-specific, can all impact sensor performance. In addition, a portion of the excitation field can couple into radiation modes and excite dipoles, while a fraction of the emitted fluorescence can also couple into radiation modes and subsequently re-couple into the fiber. By excluding these contributions, the model underestimates the total collected fluorescence. These effects were not included in the present model but will be investigated in future experimental work and extended simulations to better reflect real-world operating conditions.

## Conclusion

In this study, we developed a comprehensive theoretical model for fluorescence-based fiber optic sensors that brings together three critical aspects often neglected in earlier work: multimode excitation, incoherent emission from individually treated fluorophores, and multimode fluorescence capture. This framework provides a unified description of the complete excitation–emission–collection sequence and reveals how multimode interference shapes both fluorophore excitation and fluorescence recapture efficiency.

To demonstrate the utility of this framework, we applied it to D-shaped multimode fibers and investigated the effects of polishing depth, core size, and excitation mode on fluorescence output. The results show that polishing to approximately half the core diameter maximizes fluorescence for fundamental-mode excitation, smaller cores yield higher fluorescence than larger ones, and excitation with higher-order modes significantly enhances fluorescence output, especially in larger cores. Importantly, higher-order modes not only offset losses in large-core fibers but also enable optimal performance at shallower polishing depths, which is advantageous for fabrication and robustness.

Overall, this work provides both a general theoretical foundation for modeling fluorescence-based fiber sensors and practical design insights for D-shaped geometries. By enabling more efficient fluorescence capture under realistic multimode conditions, our framework supports the development of high-sensitivity fiber optic sensors for applications in medical diagnostics, chemical analysis, and environmental monitoring.

## Supplementary Information

Supplementary Information.

## Data Availability

Data underlying the results presented in this paper are not publicly available at this time but may be obtained from the authors upon reasonable request.
