# Supplementary material for: A rigorous theoretical model of fluorescence-based fiber optic sensors: application to D-shaped fibers
Source: Sci Rep. 2025 Dec 31;16:3331. doi: 10.1038/s41598-025-33244-8 (PMC12835182; doi:10.1038/s41598-025-33244-8)

**Supplementary Information**

**A Rigorous Theoretical Model of Fluorescence-Based Fiber Optic Sensors: Application to D-Shaped Fibers**

Shaghayegh Baghapour^1,*^, Wen Qi Zhang^1,2^, Stephen C. Warren-Smith^1,2^, Sally E. Plush^3^,

and Shahraam Afshar V.^1^

^1^Laser Physics and Photonics Devices Laboratories, STEM, Adelaide University, Adelaide, 5095, South Australia, Australia

^2^Future Industries Institute, Adelaide University, Adelaide, 5095, South Australia, Australia

^3^Clinical and Health Sciences, Adelaide University, Adelaide, 5000, South Australia, Australia

[*shaghayegh.baghapour@adelaide.edu.au](mailto:*shaghayegh.baghapour@adelaide.edu.au)

**Supplementary Figures:**

Supplementary Fig. S1:

In-coupling ($\mathrm{CE}_{\mathrm{in}}=\frac{1}{\left| a_{11}^{e} \right|^{2}}\sum_{k} \left| a_{2k}^{e} \right|^{2}$, dashed curves) and out-coupling efficiency (Eq. 19, solid curves) as a function of polishing depth for all fiber core diameters simulated in this study.


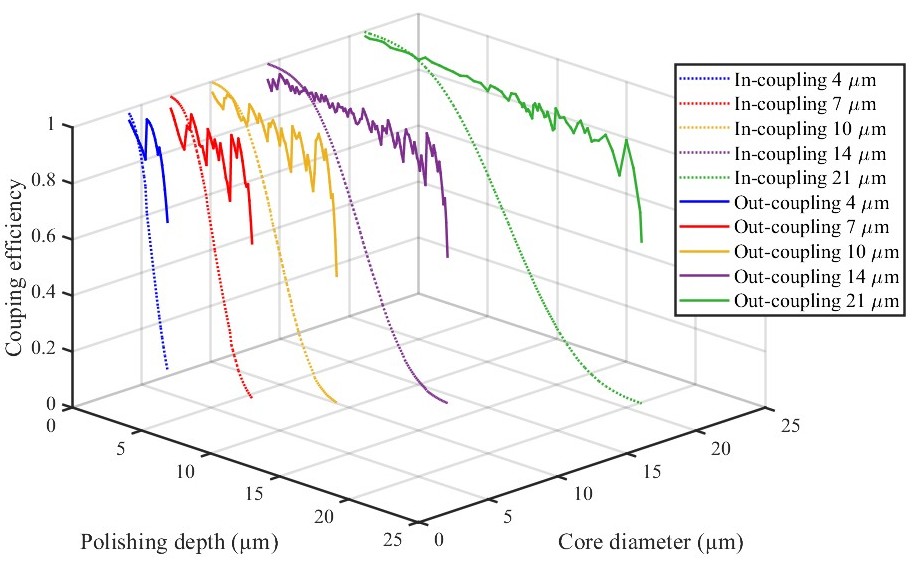

Supplement: Supplementary file 1 — Supplementary Information. [file 41598_2025_33244_MOESM1_ESM.docx]
